# Paracetamol use (and/or misuse) in children in Enugu, South-East, Nigeria

**DOI:** 10.1186/1471-2431-12-103

**Published:** 2012-07-19

**Authors:** Herbert A Obu, Josephat M Chinawa, Agozie C Ubesie, Christopher B Eke, Ikenna K Ndu

**Affiliations:** 1 Department of Paediatrics, College of Medicine, University of Nigeria, Enugu Campus & University of Nigeria Teaching Hospital, Ituku-Ozalla, Enugu, 400001, Nigeria; 2 Department of Paediatrics, University of Nigeria Teaching Hospital, Ituku-Ozalla, Enugu, Nigeria

**Keywords:** Paracetamol, Use, Misuse, Children, Caregivers

## Abstract

**Background:**

Paracetamol (also known as acetaminophen) is the commonest available analgesic and anti-pyretic. It is readily accessed from pharmacy, patent medicine and provision shops as over the counter drug making it a potential drug of abuse, especially in children. We sought to find its use and/or misuse in children seen at the paediatric outpatient clinic of the University of Nigeria Teaching Hospital (UNTH) Ituku-Ozalla, Enugu.

**Objective:**

To determine the dosage, formulation, and frequency of paracetamol administration to children by caregivers and factors associated with its use and/or misuse.

**Method:**

An observational prospective study involving 231 children and their caregivers seen at the paediatric outpatient clinic of the University of Nigeria Teaching Hospital, Ituku - Ozalla, Enugu between June and November 2011 was undertaken. Data on paracetamol use before presentation to the clinic, in addition to demographic and other data were obtained from the caregivers using a structured questionnaire. Ethical consent for the study was obtained from the Hospital Ethics and Research Committee and informed consent was further obtained from the caregivers of the children.

**Results:**

A total of 231 children aged six weeks to 16 years and their caregivers participated in this study. The mean ages of the children and their caregivers were 3.8 and 33.9 years, respectively. One hundred and thirty three of the children studied were males while 98 were females. Most of the children (75.6%) received paracetamol at home before presenting. Paracetamol tablet alone or in combination with the syrup was mostly used (60%) and this observation was made across all age groups. The commonest reason for using paracetamol tablet instead of the syrup was that it was more effective. Most caregivers relied on past experience (71.2%) rather than on enclosed information leaflet to decide the appropriate dosage. Half of the children also received other medications, mainly anti-malarials and antibiotics.

**Conclusions:**

Paracetamol was commonly given to children on “self prescription” basis and the tablet formulation was most frequently used, with the possibility of misuse and overdose. Caregivers need to be educated on age-appropriate formulations which are less likely to lead to overdose.

## Background

Paracetamol (also known as acetaminophen) is the most widely used analgesic and antipyretic [1]. It is found in many over the counter and prescription products. Given in the right dosage it is not associated with many side effects; however prolonged use may produce renal injury and massive overdose may produce hepatic injury [1]. It is the most common pharmaceutical agent involved in overdose particularly below the age of 6 years [2,3]. There is a particularly significant risk of paracetamol overdose in infants and children because of the varying dosing schedules and the variety of formulations with different strengths [3]. The easiest way to inadvertently overdose on paracetamol is to combine various cough, cold and teething medications because people are unaware that paracetamol is contained in most of them [4].

Paracetamol is a weak inhibitor of the synthesis of prostaglandins. The in vivo effects of the drug are similar to those of selective cyclooxygenase −2 inhibitors [5]. These drugs are remarkably safe in children but serious side effects can occur when used inappropriately. Liver injury secondary to repeated dosing of paracetamol should be considered when a child has received more than 75 mg/kg/day for at least 2 days or if risk factors for paracetamol toxicity exist [6].

Paracetamol abuse is said to have increased 5- folds between 1978 and 1988, and has worsened to more than 200 g/person/year in the US [7]. In the United Kingdom, the rate at which paracetamol is purchased without prescription is reported to have increased to 3500 million 500 mg tablets in the year 2000 [8]. Paracetamol overdose is a significant cause of hospital admission in the US but severe liver damage is infrequent and when it occurs the prognosis is good [8].

This study sought to assess patterns of paracetamol use and/or misuse and associated factors in children in Enugu, South-East Nigeria. To the best of our knowledge there is a dearth of published work on paracetamol use in the area and in Nigeria in general. It is hoped that this study may shed more light on the subject with a view to encouraging rational use of this common pharmaceutical agent in children.

## Methods

### Study area

The study was conducted at the paediatric outpatient clinics of the University of Nigeria Teaching Hospital (UNTH) Ituku-Ozalla, Enugu; a tertiary health institution located about 20 km from the city of Enugu in south-eastern Nigeria. The hospital receives referrals from various health facilities in Enugu State and the neighboring states of Anambra, Ebonyi, Benue, Imo and Abia.

### Study population

An observational prospective study involving 231 children aged 6 weeks to 16 years and their caregivers seen at the paediatric outpatient clinic of the University of Nigeria Teaching Hospital, Ituku-Ozalla, Enugu, between June and November 2011, was carried out using convenient sampling method. All children attending the paediatric clinic of the hospital during the period of the study and who satisfied the inclusion criteria were consecutively enrolled.

### Study procedure

A structured self administered questionnaire was used to collect information from the caregivers of children (and older children who came unaccompanied) attending the paediatric outpatient clinic of the hospital during the study period. In a few cases where the caregivers/children were illiterate, the questionnaire was administered to them by the investigators who help to clear all difficulty that may arise from filling the questionnaire. Information sought included socio-demographic characteristics, paracetamol use before presenting (including dosage, dosing interval, type of formulation used, indications, and source of prescription, etc.) and other medications given.

Efforts were made to confirm that the children actually received paracetamol, and not any other medicine, by showing the caregivers different formulations and packs of the drug used in our locale and these were easily identified.

Paracetamol over dose was assessed based on the frequency of administration or dose administered. A history of frequency of administration that exceeded four times in a 24 hour period was regarded as abuse [9]. The following dosage criteria were used to ascertain if the caregiver was giving excess dose. A dose that exceeded 10 mg/kg for children under 3 months, more than 60 mg to 120 mg (2.5 mL to 5 mL of oral suspension) for those children 3 months to 1 year of age, more than 120 mg to 250 mg (5 mL to 10 mL of oral suspension) for those aged 1 to 5 years, more than 250 mg to 500 mg for those aged 6 to 12 years of age and more than 1000 mg for those above 12 years [9].

### Inclusion criteria

Children aged between 6 weeks and 16 years and those in whom informed consent was obtained were included in the study.

### Exclusion criteria

Severely ill children Subjects and those unwilling to participate in the study were excluded.

### Data analysis

Data was analyzed with SPSS version 19. An initial frequency count of all variables was done and represented in tables. The ages and sex of the children were compared using Chi square test. Chi square test was also used to compare the relationship between age and formulation of paracetamol administered. The mean and ranges of all the variables were calculated. The level of significance was set at p ≤ 0.05.

### Ethical considerations

Ethical clearance for the study was obtained from the Ethics and Research Committee of the University of Nigeria Teaching Hospital. A written informed consent was further obtained from the caregivers and older children.

### Aim

To determine the pattern of paracetamol administration in children in Enugu, South-East Nigeria.

### Objectives

1. To determine the pattern of paracetamol use and/or misuse in children in Enugu, South-East Nigeria.

2. To determine the factors associated with paracetamol use and/or misuse in children.

## Results

### Demography

A total of 231 children were enrolled into this study. One hundred and thirty three were males while 98 were females giving a male: female ratio of 1: 0.7. The children were aged 6 weeks to 16 years. The mean age of the children was 3.8 ± 4.3 years. The most common age group in this study was under five years which represented 67.7% and 72.4% of males and females respectively. There was no statistically significant difference in age distribution of the children in relation to their sex as shown in Table 1.

**Table 1:** Age and sex distribution of the children

**Age of Clients (years)**	**Male (%)**	**Female (%)**
Less than 5	90 (67.7)	71 (72.4)
5-10	30 (22.6)	17 (17.3)
11-16	13 (9.7)	10 (10.3)
**Total**	**133 (100)**	**98 (100)**

χ² ^=^ .948, df =2, p = 0. 623.

The Caregivers were aged 18 to 57 years. Most of them 208 (90.3%) were females. The highest educational attainment of majority of the Caregivers151 (65.3%) was first degree of diploma. The rest had senior secondary 66 (28.6%), primary 7 (2.8%), junior secondary 3 (1.4%) and post graduate education 3 (1.4%), 1 (0.5%) did not indicate level of education. The various occupation of the Caregivers were civil servants 59 (25.6%), unemployed 58 (25.1%), trading 33 (14.4%), teaching 30 (13%), self-employed 18 (7.9%), health care worker 15 (6.5%), banker/marketer 9 (3.7%) and others 9 (3.8%). Among the Caregivers, 205 (88.7%) were mothers of the children, 16 (7%) were fathers, 4 (1.7%) aunts and 6 (2.6%) others (such as uncles, sisters, brothers).

#### Paracetamol use

Most of the children 175 (75.6%) received Paracetamol at home before presenting at the Clinic. Among them, the most reason for administering Paracetamol was fever 158 (68.4%). Other reasons were headache, abdominal pain or discomfort, cough as shown in Table 2.

**Table 2:** Reasons for Administering Paracetamol

**Symptom**	**Frequency (%)**
Fever	145 (62.7)
Cough and/or Catarrh	17 (7.4)
Abdominal pain/discomfort	9 (3.9)
Ear ache	8 (3.5)
Skin rash	6 (2.6)
Generalized body pains	5 (2.2)
Fast/difficulty in breathing	4 (1.7)
Convulsions	3 (1.3)
Others (injury, tooth ache, excessive crying, vomiting)	6 (2.6)
No Response	28(12.1)
Total	231

Except for two children 3 (1.1%) that received injectable paracetamol, oral administration was the preferred route 228 (98.9%). The oral formulations used, were tablet 114 (49.2%), syrup 89 (38.7%) while 28 (12.1%) received both tablet and syrup.

Among children under the age of five, syrup formulation was most commonly used 64 (39.8%) while tablet was most commonly used among those aged 5–10 years 28 (59.5%) and 11 to 16 years 12 (52.2%) as shown in Table 3. There was a statistically significant difference between the age groups of the children and formulation used.

**Table 3:** Relationship between age and formulation of Paracetamol administered

**Age (yrs)**	**Less than 5 (%)**	**5-10**	**11-16 (%)**
**Age of Clients**		**(%)**	
**(years)**			
Tablet	45(28.0)	28 (59.5)	12 (52.2)
Syrup	64 (39.8)	2 (4.3)	0 (0)
tablet and syrup	18 (11.2)	2 (4.3)	1 (4.3)
No response	34 (21.0)	15(31.9)	10(43.5)
**Total**	**161(100)**	**47(100)**	**23 (100)**

χ² ^=^ 39.2, df =4, p = 0. 00.

Almost half of the respondents (45.0%) decided on their own to administer paracetamol to their children. The rest received prescription from a doctor (20.0%), a nurse (6.5%) and from either a Patent Medicine Dealer or a Pharmacist (4.3%) as shown in Table 4. Most of them (71.2%) relied on past experience to arrive at the dose to be administered as shown in Table 5.

**Table 4:** Source of the Paracetamol Prescription

**Prescriber**	**Frequency (%)**
Doctor	46 (20.0)
Nurse	15 (6.5)
Patent Medicine Dealer/Pharmacist	10 (4.3)
Self.	104 (45.0)
No response	56 (24.2)
Total	231 (100%)

**Table 5:** Determination of dose administered

**Method of dose determination**	**Frequency (%)**
Directions on the medication	29 (26.1)
Past experience	79 (71.2)
Health care workers	3(2.7)

Paracetamol misuse was found in only a total of 4 (1.7%) cases, majority of them are aged between 6 weeks −5 years as shown in Table 6 below. Adequate dosing and frequency of paracetamol was practiced by majority of the respondent for the different age groups; however 72(31.1%) of the respondent were silent on that question.

**Table 6:** Dose and frequency of paracetamol administration distributed according to the age groups

**Dose/frequency**	**6wk < 5 yrs**	**AGE GROUPS 5–10 yrs**	**6-16 years**	**Total**
2.5 ml	34	0	0	34
≤4 times	3	0	0	3
>4 times				
5 ml	2	1	0	3
≤4 times	0	0	0	0
>4 times				
Half tablet(250 mg)	22	1	23	46
≤4 times	0	0	0	0
>4 times				
1 tablet (500 mg)	31	22	3	56
≤4 times	1	1	0	2
>4 times				
1 and 1/2 tablets	0	0	1	1
≤4 times	0	0	0	0
>4 times				
2 tablets	2	4	8	14
≤4 times	0	0	0	0
>4 times				
**Total**	**95**	**29**	**35**	**159**

## Discussion

Most of the children described in this study received paracetamol, especially to treat febrile episodes. This common practice of administering paracetamol (or other anti- pyretic) to children with fever does not appear to have any benefits from available studies [10,11]. Fever is a common symptom of childhood illness in both developed and developing countries and much time and effort is spent on attempts to reduce high temperatures in young children. Though the disease process that leads to fever may be harmful, no convincing evidence shows that fever is harmful; despite this; many parents and physician believe that antipyretic treatment improves febrile children discomfort and behavior [12]. The current guidelines of WHO on the management of fever recommends the use of paracetamol for children with a fever ≥ 39^0^ C but many a time at temperatures much lower than this value, paracetamol or other antipyretics are given in our setting. There is however insufficient data to support this practice. In a Cochrane review in which the antipyretic effect of paracetamol was compared with that of a placebo, no clear advantage of paracetamol over the placebo was demonstrated [12].

It is observed that most of the paracetamol administered to our children in this study were self prescribed with a possible tendency of misuse. The reasons for this observation are not quite clear but easy availability/accessibility of the drug, ignorance and poverty may have contributed.

Park [5] reported a growing concern over the drugs which can be given to children without prescription when parents may not be fully aware of the potential risks. It is pertinent to note that [13] in the United Kingdom the rate of paracetamol purchased without prescription is reported to have increased to 3500 million 500 mg tablets in the year 2000 [14].

Majority of the caregivers in our setting prefer tablet to syrup formulations of paracetamol. The reason for this is not obvious but the belief by some people that tablet formulations are more efficacious than syrups may be tenable here. From our knowledge of pharmacology, there should be no difference in efficacy between tablets and syrups when properly formulated. Hundie and colleagues in their study made similar observations [15].

A greater percentage of the caregivers gave paracetamol to their children via the oral route. Whereas Remsing [16] and co-workers in their series noted that parenteral paracetamol had a more rapid onset of action, however, oral formulations are safe, well absorbed and equally efficacious and should be promoted.

Majority of caregivers in our study gave paracetamol in correct doses to their children. This could be explained by the fact that most of them are educated [63%] and well informed. The use of paracetamol in therapeutic doses is generally safe although hepatotoxicity has occurred with recommended dosages in children in developing countries [16].

This study reiterates the fact that majority of childhood febrile illnesses are first treated at home using past experience as a guide. This is not surprising as no caregiver worth his salt will sit put and watch his sick child in pain and/or discomfort without doing anything. It was also suggested that it gives the child rest and sleep and enables the family to rest too [17]. In a cross-sectional survey by Ajayi et al. [18] involving 535 consecutive febrile children under 10 years of age seen in an outpatient clinic, paracetamol and chloroquine were the most commonly used drugs before presentation to the hospital. This is in consonance with our findings and underscores the need to empower caregivers by appropriate education.

## Conclusion

Paracetamol is an invaluable over-the-counter medication which is safe, readily available and affordable; though given in appropriate dose in this study, however, it has great potential for misuse and overdose.

### Recommendation

More education concerning paracetamol should be given to caregivers and older children on its appropriate dosing to avoid serious adverse events when given inappropriately.

### Limitation

A population based study or one carried out in a primary health care rather than hospital based may give a better picture of paracetamol use and/or misuse.

## Competing interest

The authors hereby declare that we have no competing interests.

## Authors’ contribution

All the authors made substantial intellectual contributions to this study. OHA was involved in the conception, design, and data collection, interpretation of results, preparation of the manuscript, revision of the article at various stages and preparation of the final draft. Other authors made substantial contributions in the design, data collection, and interpretation of the results, preparation of the manuscript, revision and preparation of the final draft. All authors read and approved the final manuscript.

## Pre-publication history

The pre-publication history for this paper can be accessed here:

http://www.biomedcentral.com/1471-2431/12/103/prepub
